# Protocol of a feasibility trial for an online group parenting intervention with an integrated mental health component for parent refugees and asylum-seekers in the United Kingdom: (LTP + EMDR G-TEP)

**DOI:** 10.1177/20503121211067861

**Published:** 2021-12-23

**Authors:** Safa Kemal Kaptan, Filippo Varese, Betul Yilmaz, Panoraia Andriopoulou, Nusrat Husain

**Affiliations:** 1 School of Health Sciences, Division of Psychology and Mental Health, Faculty of Biology, Medicine and Health, Manchester Academic Health Science Centre, The University of Manchester, Manchester, UK; 2 Department of Psychology, Manchester Metropolitan University, Manchester, UK

**Keywords:** Parenting, refugees, mental health, EMDR, remote intervention

## Abstract

**Objectives:**

Conflicts expose families to a range of factors that could have a negative impact upon parental mental health which in turn leads to poor growth and development of children. Early support can improve parental mental health and parenting behaviours but currently, there is a lack of evidence on parenting interventions for forcibly displaced populations. This study aims to deliver an online parenting intervention with a mental health component for refugee and asylum-seeker parents to evaluate its feasibility and acceptability.

**Methods:**

This is a single-arm trial without a control group. The trial aims to recruit 14 refugee and asylum-seeker parents into an Online Learning Through Play and Eye Movement Desensitization and Reprocessing Group Traumatic Episode Protocol (LTP + EMDR G-TEP). The intervention will be delivered by trained research team members using online platforms.

**Results:**

The participants’ sense of parenting competence, symptoms of traumatic stress, anxiety and depression will be measured at baseline and post-intervention. Semi-structured interviews at post-intervention will also be conducted.

**Discussion:**

This study will assess the feasibility and inform the design of a future randomized controlled trial which aims to evaluate the effectiveness of LTP + EMDR G-TEP intervention for parent refugees and asylum-seekers with young children.

## Introduction

**Figure 1.**
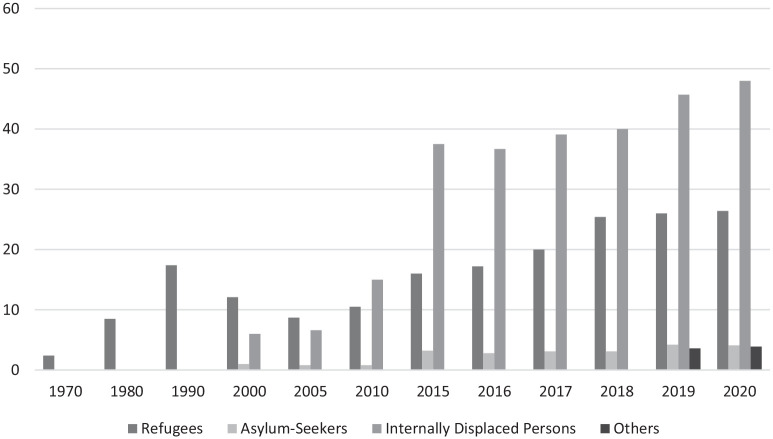
Number of displaced persons (million).

In recent years, displacement has become a significant global issue. Massive numbers of refugees, asylum-seekers and internally displaced people have been forced to leave their homes or countries due to humanitarian problems between and within nations. According to the United Nations High Commissioner for Refugees (UNHCR), the number of refugees and asylum-seekers – defined as individuals who are forcibly displaced from their country due to fear of death, conflict or human rights violations – has been rising since 2010. The most recent report by UNHCR estimates that the number of displaced people is at its highest with 82.4 million of whom 26 million were refugees and 4.1 million were asylum-seekers.^1^ Of these numbers, 40% were children under 18 years of age. Similarly, the total forcibly displaced people in the United Kingdom are also now the highest ever recorded with almost 140,000 refugees and 78,000 pending asylum applications in 2020.^2^ Figure 1 shows the worldwide trends in numbers.^1,2^

After a significant rise in the numbers, there has been increased attention to the mental health of refugees and asylum-seekers. Several studies investigated the harsh influence of war, displacement or post-migration factors on mental health and showed that these groups are likely to experience mental health difficulties including mood disorders,^3^ anxiety disorders,^4^ trauma-related disorders,^5^ psychotic disorders^6^ substance use disorders,^7^ psychoses^8^ and low levels of subjective well-being^9^ which can persist for many years.^10–16^ Moreover, the mental health difficulties appear to be a pressing problem for displaced people even after resettlement in a country that is not impacted by conflicts, as these problems are usually worsened by long resettlement processes, uncertainty or other factors in the host countries such as isolation,^17^ financial problems,^18^ acculturation issues^19^ or post-resettlement stress.^9^ Despite the acknowledgement that high levels of mental health difficulties have led to a drive towards the development of good quality services, these services are not always accessible which may worsen displaced individuals’ conditions over time.^20–22^

Mental health difficulties can be particularly devastating in the context of families. A number of studies have found that post-traumatic stress disorder (PTSD) symptoms in parent refugees are high^3,23^ and associated with harsh parenting skills and conduct problems in children including hyperactivity, emotional symptoms and peer problems^24–26^ putting family members and children in a vulnerable position.^27^ In the same vein, other studies have also shown that mental health difficulties can overburden parents which can cause withdrawal, conflict among family members and predict mental health difficulties in children.^26,28–32^ The children of these parents are also more likely to have attachment problems which generate subsequent difficulties like poor social relationships or low school performance.^33,34^

Supporting parents can facilitate a positive family environment and act as a way of reducing the risk of mental health difficulties in children. Recent studies have highlighted that parenting interventions can be accessible tools to protect family parents and children from the impact of stress and traumatic experiences, especially during the young ages when children are most sensitive to external factors.^14,35^ A considerable amount of literature has already reported the challenges that parents face in displaced contexts^36^ and highlighted the existing demand and need of caregivers for parenting skills to manage their stress and children’s behavioural and emotional changes. It is therefore obvious that parenting interventions are essential for maintaining positive parenting practices such as healthy communication and discipline strategies.^37,38^

The beneficial effects of parenting interventions in improving parenting skills in refugees or asylum-seekers have been documented across different settings.^39–41^ However, parenting interventions have been recently criticized as most of them focus solely on improving parenting knowledge or skills and do not include any strategy to address parental mental health difficulties.^42^ One way of tackling this problem is the use of holistic parenting interventions that not only improve parenting skills and knowledge but also address parental mental health difficulties.

Over the past years, there has been an increasing amount of literature on the use of online interventions^43^ which shows that online interventions in the mental health field are acceptable and produce similar outcomes to face-to-face interventions.^44,45^ The recent COVID-19 pandemic has also led to a renewed interest in online interventions. However, to our knowledge, far too little attention has been paid to this method for forcibly displaced people. The existing literature has been restricted to the use of pre-recorded video sessions or digital applications as a mode of delivery.^46^ Therefore, little is known about the feasibility of live sessions for displaced groups.

The Learning through Play (LTP) Plus Eye Movement Desensitization and Reprocessing Group Traumatic Episode Protocol (EMDR G-TEP) intervention has been planned to improve parenting satisfaction and efficacy by addressing the mental health difficulties of parent refugees and asylum-seekers with young children. The intervention combines two interventions. Component 1 is LTP that is a group parenting intervention. LTP was developed in Canada^47^ and culturally adapted versions of LTP have been used with a variety of groups including mothers with malnourished children,^48^ mothers in a low-income country^49^ and fathers with depressive symptoms.^50^ In this trial, culturally adapted LTP will be incorporated with EMDR G-TEP intervention which is a group application of widely used EMDR therapy that aims to address traumatic stress and other health difficulties.^51^ To our knowledge, this trial will be the first evaluation of the feasibility of an online-delivered parenting intervention which is a highly needed mode of delivery given the COVID-19 pandemic.

## Objectives

The main objective of the present trial is to test the feasibility and acceptability of an online-delivered LTP + EMDR G-TEP intervention along with its potential effectiveness to improve parental self-esteem and reduce mental health difficulties. This study will provide an insight into a larger randomized controlled trial (RCT) trial. This study specifically aims:

- to test the feasibility of recruiting parent refugees and asylum-seekers for an online parenting intervention with the aim of developing an RCT;
- to test the feasibility and acceptability of LTP + intervention across a range of parameters including recruitment, attendance and dropout rate. These will be improved by qualitative investigations;
- to test whether LTP+ improves parenting self-esteem and reduces stress, depression and anxiety in parent refugees and asylum-seekers compared to baseline assessment.

## Methods

The CONSORT 2010 reporting guideline – checklist for protocol of a clinical trial – was used in reporting this protocol.^52^

### Study design

The LTP+ trial is a single-arm design without a control group. The study is planned to run over 9 months and take place in the United Kingdom. Participants will receive eight sessions of LTP+ treatment and will complete a battery of questionnaires at baseline (T0) and after the treatment (T1). There is also the post-intervention qualitative part which will explore the participants’ views regarding the acceptability of the intervention. Assessment and group sessions will be done via a social platform (ZOOM). The intervention is expected to take approximately 10 weeks per participant. The research is funded by the Turkish Embassy Education Counsellor’s Office and received ethical approval from the University of Manchester Research Ethics Committee 3. The study is registered on ISRCTN (ISRCTN71853873). Table 1 presents a planned design.

**Table 1.** Design of the planned intervention.

	Study period			
	Enrolment	Baseline assessment	The intervention	Post-intervention assessment
	−*t*1	*t*0	*Eight weekly sessions*	*t*1
Enrolment:				
Eligibility screen	X			
Informed consent	X			
Intervention: LTP + EMDR G-TEP				
Assessments				
Socio-demographic questionnaire	X			
Parenting Sense of Competence Scale (PSOC)		X		X
International Trauma Questionnaire (ITQ)		X		X
Generalized Anxiety Disorder 7-item (GAD-7)		X		X
Patient Health Questionnaire-9 (PHQ-9)		X		X
Dissociative Experiences Scale-II (DES-II)		X		
Client Satisfaction Questionnaire (CSQ-8)				X
Qualitative Interview				X

EMDR G-TEP: Eye Movement Desensitization and Reprocessing Group Traumatic Episode Protocol.

### Participants

The treatment will be delivered to adult parent refugees and asylum-seekers with young children who are living in the United Kingdom. The study aims to recruit 14 participants. As this is a feasibility trial that aims to inform future randomized trials and the proposed intervention has not been studied previously, no sample size calculation was conducted. Instead, the sample size is decided in consultation with the developer of the treatment manuals and community members. Finally, previous studies with similar nature were reviewed to decide the final sample size.^38,40^ Participants will be included in the study if they are (1) aged over 18 years, (2) a biological parent or primary caregiver of young children (under the age of 3 years), (3) able to provide informed written consent and speak/read English, (4) resident in the United Kingdom as refugee or asylum-seeker and (5) registered with a General Practitioner (GP) (6) able to access to the Internet and smart devices. Participants will be excluded if they are receiving similar treatment to the planned intervention. All participants will be reimbursed for their time and effort at a rate of £10 per session.

### Intervention

LTP + EMDR G-TEP is a group parenting intervention that combines two forms of intervention: LTP and EMDR G-TEP. LTP was developed by Toronto Public Health (1993) and updated by The Hincks-Dellcrest Centre later on.^47^ The programme promotes the healthy development of children (birth to 3 years) and is using the principles of several theories including cognitive development^53^ and attachment theory.^54^ LTP aims to stimulate parent–child attachment by increasing parental participation and teaching parents how to use play activities to enhance the development of their children with the pictorial calendar. The pictorial calendar is a user-friendly tool that does not require parents to hold a high level of literacy. The calendar identifies areas where more knowledge is needed and includes culturally adapted illustrations of play activities that promote learning and attachment. The LTP programme covers the key development areas under several subheadings including a sense of self, physical development, relationships, understanding the world and communication that are written in a low-literacy language with appropriate illustrations.

In this trial, LTP intervention will be incorporated with EMDR G-TEP. G-TEP is a group intervention that aims to reduce the impact of recent traumatic experiences and process its ongoing consequences.^51^ The protocol restricts the processing of the traumatic experiences to the disturbing points to bring over quick relaxation. The protocol works on the principles of EMDR therapy^55,56^ and employs a structured worksheet with a focus on present safety and positive future templates. The worksheet is given to the participants and allows them to work on their material individually. This avoids the sharing of difficulties and disturbances, hence prevents vicarious traumatization. Participants rate the disturbance of traumatic memories during session several times to see the changes over time. Several studies tested G-TEP in various groups including refugees^57,58^ in the treatment of PTSD, depression and anxiety. Like LTP, the key component of G-TEP is the colourful worksheet that guides participants and enables quick adaptation to intervention.

### Procedure

#### Pre-intervention

Participants will be recruited via self-referrals or community referrals using snowball and convenience sampling methods. Posters and flyers will be shared with relevant organizations and displayed at places that are known to the target group. The communication list of the participants from previous studies (who have agreed to be contacted for future studies) will also be used.

Once a potentially eligible participant is identified, they will be approached by the first investigator (S.K.K.) and provided with information about the study. This will include the aims of the study, data management and confidentiality including the Participant Information Sheet (PIS). Participants will have up to 48 h to consider participation. Once participants express a wish to take part, they will be sent (posted or emailed) assessment tools along with a consent form (T0). They will also be invited to an online one-to-one baseline assessment meeting if they require further assistance in completing assessment tools. In the baseline meeting, the details of the study will be shared again, and individuals will have the opportunity to ask questions before written consent is sought. This meeting will be with just the participant and the researcher (one-to-one). The meeting will last for an hour approximately.

#### The sessions

Once the consent is received; the participants will attend weekly group sessions. The group training sessions will take place in an online platform (ZOOM) and will be delivered by the members of the research team who are clinical psychologists and trained in the treatment protocol. The facilitators will use a detailed protocol and checklist that will include continuous supervision from qualified trainers to ensure fidelity. The training will be conducted in English and delivered in eight sessions over 3 months, each session lasting about 60–90 min. The size of the groups will be six to eight individuals. Each session will focus on the stages of child development with a focus on cognitive development, physical development, communication and relationships. Starting from the third session, participants will practise EMDR G-TEP worksheet without processing any traumatic memory. The aim of these practices is to make participants familiar with the entire G-TEP protocol. Table 2 shows the list of the sessions.

**Table 2.** The list of the sessions.

**Phase**		
**Pre-**intervention	One-to-one assessment meeting	
**The intervention**	Session 1	Introduction and Ground Rules Heads Up (Birth to 2 Months)
	Session 2	The Looker (2–5 Months) The Sitter and Crawler (5–8 Months)
	Session 3	The Cruiser (8–13 Months) *Butterfly Hug
	Session 4	The Walker (13–18 Months) *Butterfly Hug
	Session 5	The Doer (18–24 Months) *4 Elements
	Session 6	The Early Tester (24–32 Months) *Safe Place
	Session 7	The Tester (32–36 Months) *Date Today
	Session 8	Disturbing Sad Memories Group Traumatic Episode Protocol
**Post-**intervention	One-to-one assessment meeting and an optional interview	

EMDR G-TEP: Eye Movement Desensitization and Reprocessing Group Traumatic Episode Protocol.

* EMDR G-TEP techniques.

Several safety and confidentiality steps will be taken. First, participants will be provided with an information sheet prior to obtaining consent to participate which will provide details of the research project. Participants will be offered the opportunity to withdraw their consent at any time during the study. All participants will be informed they will be free to withdraw from the study at any time without detriment to themselves. If a participant withdraws, the data collected up until that point will be destroyed. Finally, all participants will be placed in the waiting room before each group session and will be advised to not use their name but choose a pseudonym. If the participants would not change their names, the host will rename them before admitting them to the meeting room. Finally, the zoom sessions will be password-protected participants will be requested to not share the links and passwords with anyone else.

The risk of stress will be managed through several steps. In particular: the LTP sessions will be delivered by two facilitators. While the first facilitator will deliver the training, the second facilitator will observe participants and read signs of distress. If a participant shows any signs of distress, the co-facilitator will take the participant to a private breakout room (one-to-one) and offer one-to-one relaxation techniques such as breathing, a safe place or a four elements exercise. The final session, which is G-TEP, will be delivered by three facilitators in smaller groups of four participants to improve safety as the session will focus on stressful memories. Where participants express a need to see someone for any mental health concerns before or after the meetings, they will be referred to relevant services or their GP’s. The research team will be able to aid in doing this where the participant consents to it, for example, by contacting the organizations or their GP’s and informing them of the difficulties the participant is experiencing, and by making further referrals for support. Moreover, participants’ ability to stay within the window of tolerance will be measured using Dissociative Experiences Scale-II. This will provide information about their ability to stay within the window of tolerance, defined as an optimal zone where a participant can function effectively, and will enable facilitators to act accordingly. Finally, grounding exercises will be used to make participants to feel more here and now. The facilitators will use a detailed protocol and checklist that will include continuous supervision from qualified trainers to ensure fidelity.

#### Post-intervention

The second assessment (T1) will take place immediately after the last session of LTP + intervention. The participants will once again be posted or emailed the assessment tools and invited to one-to-one post-intervention assessment meeting. This meeting will include a semi-structured interview to explore their experiences and views on LTP+ intervention. All participants would be reimbursed for their time and effort per session.

### Data collection

#### Parenting Sense of Competence Scale–administered pre- and post-treatment

Parenting Sense of Competence Scale (PSOC)^59^ is a 17-item self-report scale to measure perceived parenting ability under two sections: efficacy and satisfaction. Section efficacy measures the ability to plan and perform the acts necessary for parenting, while section satisfaction measures the satisfaction received from parenting roles. Responses are scored on a 6-point Likert-type scale where 1 = strongly disagree, 2 = somewhat disagree, 3 = disagree, 4 = agree, 5 = somewhat agree, and 6 = strongly agree. The higher the score, the higher the sense of parenting competence. The scale has a high-level internal consistency for mothers with young children which has been reported ranging from 0.70 to 0.88.^60–64^

#### International Trauma Questionnaire–administered pre- and post-treatment

International Trauma Questionnaire (ITQ)^65^ is a self-report 18-item tool to measure traumatic stress and PTSD symptoms. Participants rate to the extent they have been distressed with its timeline. Responses are recorded on a 5-point Likert-type scale. The ITQ has received strong psychometric properties in different studies including with a sample of refugees.^65–70^

#### Generalized Anxiety Disorder 7-item–administered pre- and post-treatment

Generalized Anxiety Disorder 7-item (GAD-7)^71^ is a self-report questionnaire to assess common anxiety symptoms including generalized anxiety, panic, social anxiety, and post-traumatic stress disorders. It has a 0–3-point rating scale, providing a severity score range from 0 to 21. Good psychometric properties have been established in several studies with different sample groups including immigrants.^72–76^

#### Patient Health Questionnaire-9–administered pre- and post-treatment

Symptoms of depression will be measured using the Patient Health Questionnaire-9 (PHQ-9) tool.^77^ PHQ-9 is a 9-item self-report questionnaire to measure the severity of depression. The scale has been validated in a different setting for different populations including refugees.^78–80^

#### Dissociative Experiences Scale-II–administered pre-treatment

Dissociative Experiences Scale-II (DES-II)^81^ is a 29-item self-report questionnaire to screen dissociative experiences including amnesic experiences, imagination and depersonalization. Responses are scored on a scale of 0%–100% to indicate the frequency of dissociative experiences with a timeline of never to at least once a week. The scale has good reliability and validity for different settings, languages and sample groups.^82–84^

#### Client Satisfaction Questionnaire–administered post-treatment

The Client Satisfaction Questionnaire (CSQ-8) is among the few validated satisfaction tests used in healthcare.^85,86^ It is a brief and quick tool with only eight items. It has a 1–4-point rating scale to measure satisfaction with the provided service.

#### Feasibility and acceptability of recruitment and delivery

The feasibility of the intervention will be assessed through several variables: recruitment rate (number of participants contacted and source of recruitment), attendance and dropout rates (a record for each session and reasons for withdrawal).

#### Demographic details

A self-report questionnaire will be used to record demographic details such as resident status, time in the United Kingdom, employment, nationality, education level, marital status, age, gender, number of children, total household income, and the contact details of GP.

#### Qualitative interviews

Upon completion of the intervention, all participants will be invited to a post-intervention one-to-one semi-structured online interview to explore their experiences with the intervention. The interviews will follow topic guides to explore different aspects of the intervention including content, delivery method, usefulness, acceptability and barriers or facilitators to implementation. We will also invite participants who withdraw/drop out from the study to understand their concerns about the intervention and the perceived barriers to implementing it. The interviews will be audio-recorded and analysed using thematic analysis.^87^ Table 3 lists a summary of the topic guides.

**Table 3.** Topic guides.

Questions for all participants
What do you think about LTP + EMDR G-TEP sessions?
In what way did LTP + EMDR G-TEP had an impact on your life?
Would you recommend any changes in the LTP + EMDR G-TEP?
What was the reaction of the people around you to your attending LTP + EMDR G-TEP?
Have you faced any challenges whilst taking part in the training?
Would you recommend LTP + EMDR G-TEP to friends or family in the same situation as you?
How do you feel about the questionnaires you have completed?
Extra questions for completers
Any part of the training you are not happy with? Do you think any aspect should not be included in the training?
What aspect of the training did you find most helpful?
Extra questions for dropouts
What were your concerns regarding continuing your participation in the research?
What were the most important reasons for you to stop coming for LTP + EMDR G-TEP?
In what way did (or did not) work well for you?

LTP: Learning through Play; EMDR G-TEP: Eye Movement Desensitization and Reprocessing Group Traumatic Episode Protocol.

### Data analysis and management

#### Quantitative data

Quantitative outcome tools will be stored electronically in The University of Manchester Research Data Storage Service (Isilon). Hard copies will be stored in a locked, secure box in the locked office of the research team at the University of Manchester and digitized as soon as possible to be stored on the University of Manchester secure network in a password-protected folder. Hard copies will be destroyed post digitization. Moreover, any data generated through this project (all documents and all participants) will be given a number to protect participants’ confidentiality. The data set with the code number and identifiable data will be held separately and securely and only the research team will have access to data.

Quantitative data will be analysed using SPSS 25. Descriptive statistics will be done on the measures – baseline and post-intervention. The rating scores for all participants will be provided and compared to assess any changes over time across these scales. The mean scores, standard deviations, and ranges will also be reported. Pre- and post-test scores will be compared using the reliable change indicator analysis. Finally, descriptive statistics such as recruitment rate, attendance and dropout will be reported to document feasibility and acceptability.

#### Qualitative data

The interviews will be audio-recorded on an encrypted university-provided device that has been enrolled onto the university exchange email service to activate device encryption. Recordings will be checked once transferred to the University’s research data storage service before deleting from the recording device. Transcripts will be anonymized by removing any information that could identify the participant and replacing it with a generic description.

The interviews will be transcribed verbatim and analysed using thematic analysis.^88^ In line with the values of thematic analysis, data saturation will not be calculated.^89^ Instead, Consolidated Criteria for Reporting Qualitative (COREQ) research will be employed to ensure transparency regarding data analysis^90^ (please see the supplementary COREQ file for details of the qualitative data analysis plan).

## Discussion

Providing parents with culturally adapted interventions is crucial for a smooth transition to host countries and for managing changes in their family’s lives. One of the most important aspects of these approaches is the education of parents on the development of their children and addressing their own mental health difficulties so that they can fulfil children’s developmental potential and overcome disturbing memories of war and trauma.

It is well-documented that refugees and asylum-seekers are less engaged with mental health services than the general population despite the high prevalence of mental health difficulties.^91,92^ The weak engagement with services is further worsened by stigma and shame that are particularly relevant in face-to-face interventions.^93^ This indicates a need to explore various methods that are more creative and accessible to these groups. Online interventions can be a good response to improve accessibility and keep participants engaged with mental health interventions given the COVID-19 pandemic and other reasons specific to forcibly displaced individuals such as relocation.

The present feasibility trial is designed to evaluate an online group parenting intervention (LTP + EMDR G-TEP) for parent refugees and asylum-seekers who are residents in the United Kingdom. LTP + EMDR G-TEP intervention aims to improve parenting satisfaction and efficacy along with addressing the mental health difficulties of parents. The current trial will examine whether LTP+ intervention is acceptable, feasible and accessible when delivered in an online platform which strengthens its novelty and improves its contribution to the field.

### Limitations

This trial has several limitations. First, the challenges in accessing the Internet, smart devices or a low level of tech-literacy might pose challenges to recruitment^94^ thus might lead to a biased sample group. Second, the small sample size might limit the generalizability of the results. Third pre- and post-treatment design without a control group will weaken the evidence on the effectiveness of the intervention. Despite these limitations, the current trial will provide important insights into the feasibility of implementing online parenting interventions with a mental health component for refugees and asylum-seekers. The inclusion of qualitative interviews, however, will further improve our understanding in designing future RCT trials assessing the effectiveness of LTP+ intervention.

## Conclusion

In conclusion, to our knowledge, this is the first trial that examines the feasibility of an online parenting intervention for parent refugees and asylum-seeker. We expect that the results of this trial will provide important insight into the use of online methods for forcibly displaced people and will pave the way for future RCT trials.

## Supplemental Material

sj-doc-1-smo-10.1177_20503121211067861 – Supplemental material for Protocol of a feasibility trial for an online group parenting intervention with an integrated mental health component for parent refugees and asylum-seekers in the United Kingdom: (LTP + EMDR G-TEP) Click here for additional data file. Supplemental material, sj-doc-1-smo-10.1177_20503121211067861 for Protocol of a feasibility trial for an online group parenting intervention with an integrated mental health component for parent refugees and asylum-seekers in the United Kingdom: (LTP + EMDR G-TEP) by Safa Kemal Kaptan, Filippo Varese, Betul Yilmaz, Panoraia Andriopoulou and Nusrat Husain in SAGE Open Medicine

sj-docx-2-smo-10.1177_20503121211067861 – Supplemental material for Protocol of a feasibility trial for an online group parenting intervention with an integrated mental health component for parent refugees and asylum-seekers in the United Kingdom: (LTP + EMDR G-TEP) Click here for additional data file. Supplemental material, sj-docx-2-smo-10.1177_20503121211067861 for Protocol of a feasibility trial for an online group parenting intervention with an integrated mental health component for parent refugees and asylum-seekers in the United Kingdom: (LTP + EMDR G-TEP) by Safa Kemal Kaptan, Filippo Varese, Betul Yilmaz, Panoraia Andriopoulou and Nusrat Husain in SAGE Open Medicine

sj-docx-3-smo-10.1177_20503121211067861 – Supplemental material for Protocol of a feasibility trial for an online group parenting intervention with an integrated mental health component for parent refugees and asylum-seekers in the United Kingdom: (LTP + EMDR G-TEP) Click here for additional data file. Supplemental material, sj-docx-3-smo-10.1177_20503121211067861 for Protocol of a feasibility trial for an online group parenting intervention with an integrated mental health component for parent refugees and asylum-seekers in the United Kingdom: (LTP + EMDR G-TEP) by Safa Kemal Kaptan, Filippo Varese, Betul Yilmaz, Panoraia Andriopoulou and Nusrat Husain in SAGE Open Medicine

sj-docx-4-smo-10.1177_20503121211067861 – Supplemental material for Protocol of a feasibility trial for an online group parenting intervention with an integrated mental health component for parent refugees and asylum-seekers in the United Kingdom: (LTP + EMDR G-TEP) Click here for additional data file. Supplemental material, sj-docx-4-smo-10.1177_20503121211067861 for Protocol of a feasibility trial for an online group parenting intervention with an integrated mental health component for parent refugees and asylum-seekers in the United Kingdom: (LTP + EMDR G-TEP) by Safa Kemal Kaptan, Filippo Varese, Betul Yilmaz, Panoraia Andriopoulou and Nusrat Husain in SAGE Open Medicine

sj-docx-5-smo-10.1177_20503121211067861 – Supplemental material for Protocol of a feasibility trial for an online group parenting intervention with an integrated mental health component for parent refugees and asylum-seekers in the United Kingdom: (LTP + EMDR G-TEP) Click here for additional data file. Supplemental material, sj-docx-5-smo-10.1177_20503121211067861 for Protocol of a feasibility trial for an online group parenting intervention with an integrated mental health component for parent refugees and asylum-seekers in the United Kingdom: (LTP + EMDR G-TEP) by Safa Kemal Kaptan, Filippo Varese, Betul Yilmaz, Panoraia Andriopoulou and Nusrat Husain in SAGE Open Medicine

sj-docx-6-smo-10.1177_20503121211067861 – Supplemental material for Protocol of a feasibility trial for an online group parenting intervention with an integrated mental health component for parent refugees and asylum-seekers in the United Kingdom: (LTP + EMDR G-TEP) Click here for additional data file. Supplemental material, sj-docx-6-smo-10.1177_20503121211067861 for Protocol of a feasibility trial for an online group parenting intervention with an integrated mental health component for parent refugees and asylum-seekers in the United Kingdom: (LTP + EMDR G-TEP) by Safa Kemal Kaptan, Filippo Varese, Betul Yilmaz, Panoraia Andriopoulou and Nusrat Husain in SAGE Open Medicine

sj-docx-7-smo-10.1177_20503121211067861 – Supplemental material for Protocol of a feasibility trial for an online group parenting intervention with an integrated mental health component for parent refugees and asylum-seekers in the United Kingdom: (LTP + EMDR G-TEP) Click here for additional data file. Supplemental material, sj-docx-7-smo-10.1177_20503121211067861 for Protocol of a feasibility trial for an online group parenting intervention with an integrated mental health component for parent refugees and asylum-seekers in the United Kingdom: (LTP + EMDR G-TEP) by Safa Kemal Kaptan, Filippo Varese, Betul Yilmaz, Panoraia Andriopoulou and Nusrat Husain in SAGE Open Medicine

sj-docx-8-smo-10.1177_20503121211067861 – Supplemental material for Protocol of a feasibility trial for an online group parenting intervention with an integrated mental health component for parent refugees and asylum-seekers in the United Kingdom: (LTP + EMDR G-TEP) Click here for additional data file. Supplemental material, sj-docx-8-smo-10.1177_20503121211067861 for Protocol of a feasibility trial for an online group parenting intervention with an integrated mental health component for parent refugees and asylum-seekers in the United Kingdom: (LTP + EMDR G-TEP) by Safa Kemal Kaptan, Filippo Varese, Betul Yilmaz, Panoraia Andriopoulou and Nusrat Husain in SAGE Open Medicine

sj-docx-9-smo-10.1177_20503121211067861 – Supplemental material for Protocol of a feasibility trial for an online group parenting intervention with an integrated mental health component for parent refugees and asylum-seekers in the United Kingdom: (LTP + EMDR G-TEP) Click here for additional data file. Supplemental material, sj-docx-9-smo-10.1177_20503121211067861 for Protocol of a feasibility trial for an online group parenting intervention with an integrated mental health component for parent refugees and asylum-seekers in the United Kingdom: (LTP + EMDR G-TEP) by Safa Kemal Kaptan, Filippo Varese, Betul Yilmaz, Panoraia Andriopoulou and Nusrat Husain in SAGE Open Medicine
