# Supplementary material for: Protocol of a feasibility trial for an online group parenting intervention with an integrated mental health component for parent refugees and asylum-seekers in the United Kingdom: (LTP + EMDR G-TEP)
Source: SAGE Open Med. 2021 Dec 23;9:20503121211067861. doi: 10.1177/20503121211067861 (PMC8724986; doi:10.1177/20503121211067861)
Supplement: sj-docx-2-smo-10.1177_20503121211067861 – Supplemental material for Protocol of a feasibility trial for an online group parenting intervention with an integrated mental health component for parent refugees and asylum-seekers in the United Kingdom: (LTP + EMDR G-TEP) [file sj-docx-2-smo-10.1177_20503121211067861.docx]

Consolidated criteria for reporting qualitative studies (COREQ): 32- item checklist

Tong A, Sainsbury P, Craig J. Consolidated criteria for reporting qualitative research (COREQ): a 32-item checklist for interviews and focus groups. International Journal for Quality in Health Care. 2007. Volume 19, Number 6: pp. 349 – 357

| **No. Item** | **Guide**  **questions/description** |  |
| --- | --- | --- |
| **Domain 1: Research team**  **and reﬂexivity** |  |  |
| **Personal**  **Characteristics** |  |  |
| 1.  Interviewer/facilitator | Which author/s conducted the interview or focus group? | *Interviews will be conducted by the two members of the research team: SKK, PA* |
| 2. Credentials | What were the researcher’s credentials? E.g. PhD, MD | *Safa Kemal Kaptan, MA Panoraia Andriopoulou, PhD* |
| 3. Occupation | What was their occupation at the time of the study? | *Safa Kemal Kaptan: researcher at the University of Manchester*  *Panoraia Andriopoulou: a HCPC registered Clinical & Counselling Psychologist and Senior Lecturer in Psychology at Manchester Metropolitan University* |
| 4. Gender | Was the researcher male or female? | *Safa Kemal Kaptan; Male Panoraia Andriopoulou: Female* |
| 5. Experience and training | What experience or training did the researcher have? | *Both researchers have previously participated in several qualitative studies and performed thematic analysis.* |
| **Relationship with**  **participants** |  |  |
| 6. Relationship established | Was a relationship  established prior to study commencement? | *The research team has no pre-existing relationship with the participants.* |
| 7. Participant knowledge of the interviewer | What did the participants know about the researcher? e.g. personal goals, reasons for doing  the research | *Before commencing the sessions or an interview, participants will be informed of the aims and motivations for doing the study.* |
| 8. Interviewer characteristics | What characteristics were reported about the interviewer/facilitator? e.g. Bias, assumptions, reasons and interests in the research  topic | *Participants will know that the research team is interested in parenting interventions and the mental health of disadvantaged communities. No other interviewer-related biases identified.* |

| **Domain 2: study design** |  |  |
| --- | --- | --- |
| **Theoretical framework** |  |  |
| 9. Methodological orientation and Theory | What methodological orientation was stated to underpin the study? e.g. grounded theory, discourse analysis, ethnography, phenomenology, content  analysis | *Thematic analysis* |
| **Participant selection** |  |  |
| 10. Sampling | How were participants selected? e.g. purposive, convenience, consecutive,  snowball | *Participants who agreed to take part in intervention will be invited to post-intervention interviews.* |
| 11. Method of approach | How were participants  approached? e.g. face- to- face, telephone, mail, email | *Email* |
| 12. Sample size | How many participants were  in the study? | *All participants (N=14) will be invited* |
| 13. Non-participation | How many people refused to participate or dropped out?  Reasons? | *N/A* |
| Setting |  |  |
| 14. Setting of data collection | Where was the data  collected? e.g. home, clinic, workplace | *Data will be collected via ZOOM* |
| 15. Presence of non- participants | Was anyone else present besides the participants and  researchers? | *No* |
| 16. Description of sample | What are the important characteristics of the sample? e.g. demographic  data, date | *Participants will be adult refugees and asylum seekers with young children.* |
| Data collection |  |  |
| 17. Interview guide | Were questions, prompts,  guides provided by the authors? Was it pilot tested? | *As the interviews proceeded, the interview guide will be updated* |
| 18. Repeat interviews | Were repeat interviews  carried out? If yes, how many? | *No* |
| 19. Audio/visual recording | Did the research use audio or visual recording to collect  the data? | *Data will be audio recorded using a digital recorder* |
| 20. Field notes | Were ﬁeld notes made during and/or after the inter  view or focus group? | *No* |
| 21. Duration | What was the duration of the  interviews or focus group? | *The expected duration is an hour* |
| 22. Data saturation | Was data saturation discussed? | *Qualitative data will be analyzed using thematic*  *analyses. This approach argues that the idea of saturation is not consistent with the values and* |

|  |  | *assumptions of thematic analyses.* |
| --- | --- | --- |
| 23. Transcripts returned | Were transcripts returned to participants for comment  and/or correction? | *Yes it will be returned to participants* |
| **Domain 3: analysis and**  **ﬁndings** |  |  |
| Data analysis |  |  |
| 24. Number of data coders | How many data coders  coded the data? | *Two* |
| 25. Description of the coding tree | Did authors provide a  description of the coding tree? | *Thematic map will be added* |
| 26. Derivation of themes | Were themes identiﬁed in advance or derived from the  data? | *Themes will be generated from the data* |
| 27. Software | What software, if applicable, was used to  manage the data? | *Nvivo* |
| 28. Participant checking | Did participants provide feedback on the  ﬁndings? | *Yes, feedback will be provided* |
| Reporting |  |  |
| 29. Quotations presented | Were participant quotations presented to illustrate the  themes/ﬁndings? Was each quotation identiﬁed? e.g.  participant number | *Yes* |
| 30. Data and ﬁndings consistent | Was there consistency between the data  presented and the ﬁndings? | *N/A* |
| 31. Clarity of major themes | Were major themes clearly  presented in the ﬁndings? | *N/A* |
| 32. Clarity of minor themes | Is there a description of diverse cases or discussion  of minor themes? | *N/A* |
