# Supplementary material for: Protocol of a feasibility trial for an online group parenting intervention with an integrated mental health component for parent refugees and asylum-seekers in the United Kingdom: (LTP + EMDR G-TEP)
Source: SAGE Open Med. 2021 Dec 23;9:20503121211067861. doi: 10.1177/20503121211067861 (PMC8724986; doi:10.1177/20503121211067861)
Supplement: sj-docx-3-smo-10.1177_20503121211067861 – Supplemental material for Protocol of a feasibility trial for an online group parenting intervention with an integrated mental health component for parent refugees and asylum-seekers in the United Kingdom: (LTP + EMDR G-TEP) [file sj-docx-3-smo-10.1177_20503121211067861.docx]

Dissociative Experiences Scale - II

*This questionnaire asks about experiences that you may have in your daily life. We are interested in how often you have these experiences. It is important, however that your answers show how often these experiences happen to you when you are not under the influence of alcohol or drugs. To answer the questions, please determine to what degree each experience described in the question applies to you and circle the number to shoe what percentage of the time you have the experience.*

*Carlson, E.B. & Putnam, F.W. (1993). An update on the Dissociative Experience Scale. Dissociation 6(1), p. 16-27.*

***For example: 0% (Never) 10 20 30 40 50 60 70 80 90 100% (Always)***

1. Some people have the experience of driving or riding in a car or bus or subway and suddenly realizing that they don’t remember what has happened during all or part of the trip. Circle the number to show what percentage of the this happens to you.

0% 10 20 30 40 50 60 70 80 90 100%

1. Some people find that sometimes they are listening to someone talk and they suddenly realize that they did not hear part or all of what was said. Circle the number to show what percentage of the this happens to you.

0% 10 20 30 40 50 60 70 80 90 100%

1. Some people have the experience of finding themselves in a place and have no idea how they got there. Circle the number to show what percentage of the this happens to you.

0% 10 20 30 40 50 60 70 80 90 100%

1. Some people have the experience of finding themselves dressed in clothes that they don’t

remember putting on. Circle the number to show what percentage of the this happens to you.

0% 10 20 30 40 50 60 70 80 90 100%

1. Some people have the experience of finding new things among their belongings that they do not remember buying. Circle the number to show what percentage of the this happens to you.

0% 10 20 30 40 50 60 70 80 90 100%

1. Some people sometimes find that they are approached by people that they do not know, who call them by another name or insist that they have them before. Circle the number to show what percentage of the this happens to you.

0% 10 20 30 40 50 60 70 80 90 100%

1. Some people sometimes have the experience of feelings as though they are standing next to themselves or watching themselves do something and they actually see themselves as if they were looking another person. Circle the number to show what percentage of the this happens to you.

0% 10 20 30 40 50 60 70 80 90 100%

1. Some people are told that they sometimes do not recognize friends of family members. Circle the number to show what percentage of the this happens to you.

0% 10 20 30 40 50 60 70 80 90 100%

1. Some people find that they have no memory for some important events in their lives (for example, a wedding or graduation). Circle the number to show what percentage of the this happens to you.

0% 10 20 30 40 50 60 70 80 90 100%

1. Some people have the experience of being accused of lying when they do not think that they have lied. Circle the number to show what percentage of the this happens to you.

0% 10 20 30 40 50 60 70 80 90 100%

1. Some people have the experience of looking in a mirror and not recognizing themselves. Circle the number to show what percentage of the this happens to you.

0% 10 20 30 40 50 60 70 80 90 100%

1. Some people have the experience of feeling that other people, objects, and the world around them are not real. Circle the number to show what percentage of the this happens to you.

0% 10 20 30 40 50 60 70 80 90 100%

1. Some people have the experience of feeling that their body does not seem to belong to them. Circle the number to show what percentage of the this happens to you.

0% 10 20 30 40 50 60 70 80 90 100%

1. Some people have the experience of sometimes remembering a past event so vividly that they feel as if they were reliving that event. Circle the number to show what percentage of the this happens to you.

0% 10 20 30 40 50 60 70 80 90 100%

1. Some people have the experience of not being sure whether things that they remember happening really did happen or whether they just dreamed them. Circle the number to show what percentage of the this happens to you.

0% 10 20 30 40 50 60 70 80 90 100%

1. Some people have the experience of being in a familiar place but finding it strange and unfamiliar. Circle the number to show what percentage of the this happens to you.

0% 10 20 30 40 50 60 70 80 90 100%

1. Some people find that when they are watching television or a movie, they become so absorbed in the story that they are unaware of other events happening around them. Circle the number to show what percentage of the this happens to you.

0% 10 20 30 40 50 60 70 80 90 100%

1. Some people find that they become so involved in a fantasy or daydream that it feels as though it were really happening to them. Circle the number to show what percentage of the this happens to you.

0% 10 20 30 40 50 60 70 80 90 100%

1. Some people find that they sometimes are able to ignore pain. Circle the number to show what percentage of the this happens to you.

0% 10 20 30 40 50 60 70 80 90 100%

1. Some people find that the sometimes sit staring off into space, thinking nothing, and are not aware of the passage of time. Circle the number to show what percentage of the this happens to you.

0% 10 20 30 40 50 60 70 80 90 100%

1. Some people sometimes find that when they are alone, they talk out loud to themselves. Circle the number to show what percentage of the this happens to you.

0% 10 20 30 40 50 60 70 80 90 100%

1. Some people find that in one situation they may act so differently compared with another situation that they feel almost as if they two different people. Circle the number to show what percentage of the this happens to you.

0% 10 20 30 40 50 60 70 80 90 100%

1. Some people sometimes find that in certain situation they are able to do things with amazing ease and spontaneity that would usually be difficult for them. (for example, sports, work, social situation, etc.) Circle the number to show what percentage of the this happens to you.
2. S have j letter happe

| 0% 10 20 30 40 50 60 70 80 90 100%  ome people sometimes find that they cannot remember wh ust thought about doing that thing (for example, not knowin or have just though about mailing it). Circle the number to ns to you.  0% 10 20 30 40 50 60 70 80 90 100% |
| --- |
| ome people find evidence that they have done things they r to show what percentage of the this happens to you. |
| 0% 10 20 30 40 50 60 70 80 90 100% |
| ome people sometimes find writing, drawings, or notes amon ut cannot remember doing. Circle the number to show what |
| 0% 10 20 30 40 50 60 70 80 90 100% |
| ome people sometimes find that they hear voices inside the ent on thing that they are doing. Circle the number to show w |
| 0% 10 20 30 40 50 60 70 80 90 100% |

ether they have done something or g whether they have just mailed a show what percentage of the this

1. S numbe

do not remember doing. Circle the

1. S done b

g their belongings that they must have percentage of the this happens to you.

1. S com you.

r head that tell them to do things or hat percentage of the this happens to

1. Some people sometimes feel as if they are looking at the world through a fog, so that people and objects appear far away or unclear. Circle the number to show what percentage of the this happens to you.

0% 10 20 30 40 50 60 70 80 90 100%
