# Supplementary material for: Protocol of a feasibility trial for an online group parenting intervention with an integrated mental health component for parent refugees and asylum-seekers in the United Kingdom: (LTP + EMDR G-TEP)
Source: SAGE Open Med. 2021 Dec 23;9:20503121211067861. doi: 10.1177/20503121211067861 (PMC8724986; doi:10.1177/20503121211067861)
Supplement: sj-docx-4-smo-10.1177_20503121211067861 – Supplemental material for Protocol of a feasibility trial for an online group parenting intervention with an integrated mental health component for parent refugees and asylum-seekers in the United Kingdom: (LTP + EMDR G-TEP) [file sj-docx-4-smo-10.1177_20503121211067861.docx]

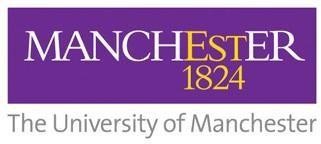


# Generalized Anxiety Disorder 7-item (GAD-7) Scale

Mossman, S. A., Luft, M. J., Schroeder, H. K., Varney, S. T., Fleck, D. E., Barzman, D. H., Gilman, R., DelBello, M. P., & Strawn, J. R. (2017). The Generalized Anxiety Disorder 7-item scale in adolescents with generalized anxiety disorder: Signal detection and validation. *Annals of clinical psychiatry : official journal of the American Academy of Clinical Psychiatrists*, *29*(4), 227–234A.

# Over the last 2 weeks, how often have you been bothered by any of the following problems?

*(Use “*✔*” to indicate your answer)*

|  |  | **Not at all** | **Several days** | **More than half the days** | **Nearly every day** |
| --- | --- | --- | --- | --- | --- |
| 1 | Feeling nervous, anxious, or on edge | 0 | 1 | 2 | 3 |
| 2 | Not being able to stop or control worrying | 0 | 1 | 3 | 3 |
| 3 | Worrying too much about different things | 0 | 1 | 3 | 3 |
| 4 | Trouble relaxing | 0 | 1 | 3 | 3 |
| 5 | Being so restless that it's hard to sit still | 0 | 1 | 3 | 3 |
| 6 | Becoming easily annoyed or irritable | 0 | 1 | 3 | 3 |
| 7 | Feeling afraid as if something awful might happen | 0 | 1 | 3 | 3 |

If you checked off any problems, how difficult have these made it for you to do your work, take care of things at home, or get along with other people?

Not difficult at all : Somewhat difficult : Very difficult : Extremely difficult :

Version 2: 24.01.2021 Page 1
