# Supplementary material for: Protocol of a feasibility trial for an online group parenting intervention with an integrated mental health component for parent refugees and asylum-seekers in the United Kingdom: (LTP + EMDR G-TEP)
Source: SAGE Open Med. 2021 Dec 23;9:20503121211067861. doi: 10.1177/20503121211067861 (PMC8724986; doi:10.1177/20503121211067861)
Supplement: sj-docx-5-smo-10.1177_20503121211067861 – Supplemental material for Protocol of a feasibility trial for an online group parenting intervention with an integrated mental health component for parent refugees and asylum-seekers in the United Kingdom: (LTP + EMDR G-TEP) [file sj-docx-5-smo-10.1177_20503121211067861.docx]

International Trauma Questionnaire

Cloitre, M, Shevlin, M, Brewin, CR, Bisson, JI, Roberts, NP, Maercker, A, Karatzias, T, Hyland, P. The International

Trauma Questionnaire: development of a self‐report measure of ICD‐11 PTSD and complex PTSD.

# Please identify the experience that troubles you most and answer the questions in relation to this experience.

**Brief description of the experience**

# When did the experience occur? (circle one)

- less than 6 months ago
- 6 to 12 months ago
- 1 to 5 years ago
- to 10 years ago
- 10 to 20 years ago
- more than 20 years ago

Below are a number of problems that people sometimes report in response to traumatic or stressful life events. Please read each item carefully, then circle one of the numbers to the right to indicate how much you have been bothered by that problem in the past month.

|  | **Not at all** | **A little bit** | **Moderat ely** | **Quite a bit** | **Extremel y** |
| --- | --- | --- | --- | --- | --- |
| P1. Having upsetting dreams that replay part of the experience or are clearly related to the experience? | 0 | 1 | 2 | 3 | 4 |
| P2. Having powerful images or memories that sometimes come into your mind in which you feel the experience is happening again in the here and now? | 0 | 1 | 2 | 3 | 4 |
| P3. Avoiding internal reminders of the experience (for example, thoughts, feelings, or physical sensations)? | 0 | 1 | 2 | 3 | 4 |
| P4. Avoiding external reminders of the experience (for example, people, places, conversations, objects,  activities, or situations)? | 0 | 1 | 2 | 3 | 4 |
| P5. Being “super-alert”, watchful, or on guard? | 0 | 1 | 2 | 3 | 4 |
| P6. Feeling jumpy or easily startled? | 0 | 1 | 2 | 3 | 4 |
| ***In the past month have the above problems:*** | | | | | |
| P7. Affected your relationships or social life? | 0 | 1 | 2 | 3 | 4 |
| P8. Affected your work or ability to work? | 0 | 1 | 2 | 3 | 4 |
| P9. Affected any other important part of your life such as parenting, or school or college work, or other important activities? | 0 | 1 | 2 | 3 | 4 |

Below are problems that people who have had stressful or traumatic events sometimes experience. The questions refer to ways you typically feel, ways you typically think about yourself and ways you typically relate to others. Answer the following thinking about how true each statement is of you.

| ***How true is this of you?*** | **Not**  **at all** | **A little**  **bit** | **Moderat**  **ely** | **Quite**  **a bit** | **Extremel**  **y** |
| --- | --- | --- | --- | --- | --- |
| C1. When I am upset, it takes me a long time to calm down. | 0 | 1 | 2 | 3 | 4 |
| C2. I feel numb or emotionally shut down. | 0 | 1 | 2 | 3 | 4 |
| C3. I feel like a failure. | 0 | 1 | 2 | 3 | 4 |
| C4. I feel worthless. | 0 | 1 | 2 | 3 | 4 |
| C5. I feel distant or cut off from people. | 0 | 1 | 2 | 3 | 4 |
| C6. I find it hard to stay emotionally close to people. | 0 | 1 | 2 | 3 | 4 |

***In the past month, have the above problems in emotions, in beliefs about yourself and in relationships:***

| C7. Created concern or distress about your relationships or social life? | 0 | 1 | 2 | 3 | 4 |
| --- | --- | --- | --- | --- | --- |
| C8. Affected your work or ability to work? | 0 | 1 | 2 | 3 | 4 |
| C9. Affected any other important parts of your life such as parenting, or school or college work, or  other important activities? | 0 | 1 | 2 | 3 | 4 |
