# Supplementary material for: Protocol of a feasibility trial for an online group parenting intervention with an integrated mental health component for parent refugees and asylum-seekers in the United Kingdom: (LTP + EMDR G-TEP)
Source: SAGE Open Med. 2021 Dec 23;9:20503121211067861. doi: 10.1177/20503121211067861 (PMC8724986; doi:10.1177/20503121211067861)
Supplement: sj-docx-6-smo-10.1177_20503121211067861 – Supplemental material for Protocol of a feasibility trial for an online group parenting intervention with an integrated mental health component for parent refugees and asylum-seekers in the United Kingdom: (LTP + EMDR G-TEP) [file sj-docx-6-smo-10.1177_20503121211067861.docx]

Parenting Sense of Competence Scale

[Ohan, J. L., Leung, D. W., & Johnston, C. (2000). The Parenting Sense of Competence Scale: Evidence of a stable](https://www.researchgate.net/profile/Jeneva_Ohan/publication/232544864_The_Parenting_Sense_of_Competence_scale_Evidence_of_a_stable_factor_structure_and_validity/links/02e7e529fe6538069a000000.pdf) [factor structure and validity. Canadian Journal of Behavioural Science/Revue canadienne des Sciences](https://www.researchgate.net/profile/Jeneva_Ohan/publication/232544864_The_Parenting_Sense_of_Competence_scale_Evidence_of_a_stable_factor_structure_and_validity/links/02e7e529fe6538069a000000.pdf) [du comportement, 32(4), 251-261.](https://www.researchgate.net/profile/Jeneva_Ohan/publication/232544864_The_Parenting_Sense_of_Competence_scale_Evidence_of_a_stable_factor_structure_and_validity/links/02e7e529fe6538069a000000.pdf)

Please rate the extent to which you agree or disagree with each of the following statements.

| **Strongly**  **Disagree** | **Somewhat**  **Disagree** | **Disagree** | **Agree** | **Somewhat**  **Agree** | **Strongly**  **Agree** |
| --- | --- | --- | --- | --- | --- |
| **1** | **2** | **3** | **4** | **5** | **6** |

| 1 | The problems of taking care of a child are easy to solve once you know | 1 | 2 | 3 | 4 | 5 | 6 |
| --- | --- | --- | --- | --- | --- | --- | --- |
|  | how your actions affect your child, an understanding I have acquired. |  |  |  |  |  |  |
| 2 | Even though being a parent could be rewarding, I am frustrated now | 1 | 2 | 3 | 4 | 5 | 6 |
|  | while my child is at his / her present age. |  |  |  |  |  |  |
| 3 | I go to bed the same way I wake up in the morning, feeling I have not | 1 | 2 | 3 | 4 | 5 | 6 |
|  | accomplished a whole lot. |  |  |  |  |  |  |
| 4 | I do not know why it is, but sometimes when I’m supposed to be in | 1 | 2 | 3 | 4 | 5 | 6 |
|  | control, I feel more like the one being manipulated. |  |  |  |  |  |  |
| 5 | My mother was better prepared to be a good mother than I am. | 1 | 2 | 3 | 4 | 5 | 6 |
| 6 | I would make a fine model for a new mother to follow in order to | 1 | 2 | 3 | 4 | 5 | 6 |
|  | learn what she would need to know in order to be a good parent. |  |  |  |  |  |  |
| 7 | Being a parent is manageable, and any problems are easily solved. | 1 | 2 | 3 | 4 | 5 | 6 |
| 8 | A difficult problem in being a parent is not knowing whether you’re | 1 | 2 | 3 | 4 | 5 | 6 |
|  | doing a good job or a bad one. |  |  |  |  |  |  |
| 9 | Sometimes I feel like I’m not getting anything done. | 1 | 2 | 3 | 4 | 5 | 6 |
| 10 | I meet by own personal expectations for expertise in caring for my child. | 1 | 2 | 3 | 4 | 5 | 6 |
| 11 | If anyone can find the answer to what is troubling my child, I am the one | 1 | 2 | 3 | 4 | 5 | 6 |
| 12 | My talents and interests are in other areas, not being a parent. | 1 | 2 | 3 | 4 | 5 | 6 |
| 13 | Considering how long I’ve been a mother, I feel thoroughly familiar with | 1 | 2 | 3 | 4 | 5 | 6 |
|  | this role. |  |  |  |  |  |  |
| 14 | If being a mother of a child were only more interesting, I would be | 1 | 2 | 3 | 4 | 5 | 6 |
|  | motivated to do a better job as a parent. |  |  |  |  |  |  |
| 15 | I honestly believe I have all the skills necessary to be a good mother to | 1 | 2 | 3 | 4 | 5 | 6 |
|  | my child. |  |  |  |  |  |  |
| 16 | Being a parent makes me tense and anxious. | 1 | 2 | 3 | 4 | 5 | 6 |
| 17 | Being a good mother is a reward in itself. | 1 | 2 | 3 | 4 | 5 | 6 |

Version 2: 24.01.2021 Page 1
