# Supplementary material for: Protocol of a feasibility trial for an online group parenting intervention with an integrated mental health component for parent refugees and asylum-seekers in the United Kingdom: (LTP + EMDR G-TEP)
Source: SAGE Open Med. 2021 Dec 23;9:20503121211067861. doi: 10.1177/20503121211067861 (PMC8724986; doi:10.1177/20503121211067861)
Supplement: sj-docx-7-smo-10.1177_20503121211067861 – Supplemental material for Protocol of a feasibility trial for an online group parenting intervention with an integrated mental health component for parent refugees and asylum-seekers in the United Kingdom: (LTP + EMDR G-TEP) [file sj-docx-7-smo-10.1177_20503121211067861.docx]

Patient Health Questionnaire-9

Bernd Löwe, Unützer, J., Callahan, C., Perkins, A., & Kroenke, K. (2004). Monitoring Depression Treatment Outcomes with the Patient Health Questionnaire-9. *Medical Care, 42*(12), 1194-1201.

**Over the last 2 weeks, how often have you been bothered by any of the following problems?**

*(Use “*✔*” to indicate your answer)*

|  |  | **Not at all** | **Several** | **More** | **Nearly** |
| --- | --- | --- | --- | --- | --- |
|  |  |  | **days** | **than half** | **every** |
|  |  |  |  | **the days** | **day** |
| 1 | Little interest or pleasure in doing things | 0 | 1 | 2 | 3 |
| 2 | Feeling down, depressed, or hopeless | 0 | 1 | 2 | 3 |
| 3 | Trouble falling or staying asleep, or sleeping too | 0 | 1 | 2 | 3 |
|  | much |  |  |  |  |
| 4 | Feeling tired or having little energy | 0 | 1 | 2 | 3 |
| 5 | Poor appetite or overeating | 0 | 1 | 2 | 3 |
| 6 | Feeling bad about yourself — or that you are a | 0 | 1 | 2 | 3 |
|  | failure or have let yourself or your family down |  |  |  |  |
| 7 | Trouble concentrating on things, such as reading | 0 | 1 | 2 | 3 |
|  | the newspaper or watching television |  |  |  |  |
| 8 | Moving or speaking so slowly that other people | 0 | 1 | 2 | 3 |
|  | could have noticed? Or the opposite — being so |  |  |  |  |
|  | fidgety or restless that you have been moving |  |  |  |  |
|  | around a lot more than usual |  |  |  |  |
| 9 | Thoughts that you would be better off dead or of | 0 | 1 | 2 | 3 |
|  | hurting yourself in some way |  |  |  |  |

If you checked off any problems, how difficult have these made it for you to do your work, take care of things at home, or get along with other people?

| Not difficult at all | : |
| --- | --- |
| Somewhat difficult | : |
| Very difficult | : |
| Extremely difficult | : |

Version 2: 24.01.2021 Page 1
