# Supplementary material for: Protocol of a feasibility trial for an online group parenting intervention with an integrated mental health component for parent refugees and asylum-seekers in the United Kingdom: (LTP + EMDR G-TEP)
Source: SAGE Open Med. 2021 Dec 23;9:20503121211067861. doi: 10.1177/20503121211067861 (PMC8724986; doi:10.1177/20503121211067861)
Supplement: sj-docx-9-smo-10.1177_20503121211067861 – Supplemental material for Protocol of a feasibility trial for an online group parenting intervention with an integrated mental health component for parent refugees and asylum-seekers in the United Kingdom: (LTP + EMDR G-TEP) [file sj-docx-9-smo-10.1177_20503121211067861.docx]

Feasibility Trial of a parenting intervention with mental health component (LTP + EMDR G-TEP)

# Qualitative research Topic Guide

**Preamble**

Thank for agreeing to take part in this interview. Introduce the study and aims of the interview.

# Participants

1. What do you think about the LTP+ EMDR G-TEP sessions?

***Prompts:*** How acceptable were they? Easy/difficult to understand?

1. In what way LTP+ EMDR G-TEP had an impact on your life?

***Prompts:*** Effects on personal life, Family life, Relationships, Mood, Attitude/behaviour, Way of thinking

1. Would you recommend any changes in the LTP+ EMDR G-TEP?

***Prompts:*** Length, number of sessions, group size, the content

1. What was the reaction of the people around you to your attending LTP+ EMDR G-TEP? (did people know?)

***Prompts:*** Family, Relatives, Friends

1. What aspect of the training did you find most helpful?

***Prompts:*** Worked on thoughts? Parenting skills? G-TEP sheet

1. Any part of the training you are not happy with? You think any aspect that should not be included in the training?

***Prompts***: Pictorial calendar, G-TEP worksheet etc.

1. Have you faced any challenges whilst taking part in the training?

***Prompts:*** Stigma, Time constraints, Family pressure, technology

1. How do you feel about the questionnaires you have completed?

***Prompts:*** Language, time takes to complete, Number of the questions

1. What are your thoughts about online delivery? Was it easy to access or use?

***Prompts:*** Access to smart-devices, internet, technological literacy

1. Would you recommend LTP+ EMDR G-TEP to friends or family in the same situation as you?

Version 2; Date: 23.01.2021 Page 1
